# Genomic, structural, and molecular analysis of calmodulin-binding transcriptional activators (CAMTAs) suggests their role in plant development and abiotic stress tolerance in chickpea

**DOI:** 10.1016/j.csbj.2025.08.032

**Published:** 2025-08-29

**Authors:** Kamankshi Sonkar, Saravanappriyan Kamali, Atul Kumar, Deepika Deepika, Ankit Ankit, Amarjeet Singh

**Affiliations:** BRIC- National Institute of Plant Genome Research, New Delhi 110067, India

**Keywords:** Calmodulin, CAMTA, Expression, Structure, Abiotic stress, Development, Regulon

## Abstract

The calmodulin-binding transcriptional activator (CAMTA) transcription factors regulate the expression of target genes in Ca^2 +^ dependent cellular functions. CAMTAs are known to regulate biotic and abiotic stress tolerance, and development in plants. CAMTA family has been characterized in Arabidopsis, it is yet to be explored in the legume plant chickpea. Here, we have identified and characterized the chickpea CAMTA family. Total seven *CAMTA* genes (*CaCAMTA1–7*) were identified in chickpea. Gene and domain structure analyses suggested that CAMTAs are structurally conserved. The phylogenetic analysis demarcated CaCAMTAs into three groups namely; group I, II and III, and indicated that CaCAMTAs have co-evolved in dicot leguminous plants whereas, they have divergent evolution in monocots. Protein homology modeling revealed their three-dimensional structure, and composition & conformations of α-helix, β-sheets and p-loops. Subcellular localization showed that CaCAMTA4 was localized both, in the nucleus and the cytosol whereas, CaCAMTA5 was localized in the nucleus. CaCAMTA promoters contain various *cis*-regulatory elements related to abiotic stresses and plant development. Expression profiling using RNA-seq data revealed differential expression of CaCAMTAs during various stages of plant development. RT-qPCR expression analysis showed that most *CaCAMTA* genes are drought, salt, and ABA responsive, suggesting their role in abiotic stress tolerance in chickpea. Moreover, CaCAMTA regulon was identified based on the presence of CAMTA binding motif (CGCG box) in the promoters of target genes, and *in-silico* interaction analysis of TF and putative targets. Overall, CaCAMTAs are crucial for abiotic stress tolerance and plant development in chickpea. Key *CaCAMTA* genes will be functionally characterized, and will be exploited for developing stress tolerant chickpea varieties.

## Graphical Abstract

**Figure:**
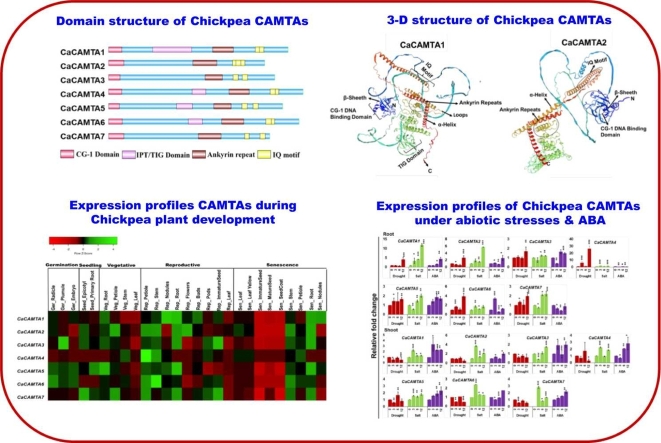

## 1 Introduction

Chickpea is one of the most important food legumes, and it is consumed worldwide. India is the largest contributor to world’s chickpea production with an annual production of about 13.75 million tonnes (mt) in the year 2021–22 [1]. Richness of chickpea seed in crude protein, carbohydrate, and essential minerals makes it an important part of vegetarian diet [2]. Unfortunately, due to harsh environmental conditions chickpea production suffers significant yield losses. Abiotic stresses, like water deficit, high soil salinity, and temperature extremes are the main despoilers, and together they account for more than 60 % annual chickpea yield losses worldwide. Particularly, the onslaught of abiotic stresses during reproductive developmental stages of chickpea, including flower set, pollen development, and pod set/abortion adversely affects the chickpea yield. Thus, abiotic stresses are damaging to the chickpea productivity. Hence, identification and characterization of potential genes for imparting abiotic stress resilience in chickpea in the era of drastic climate change is desired.

Calcium (Ca^2+^) is an important secondary messenger in the intracellular signaling, and it is involved in diverse developmental and environmental responses in plants [4], [3]. Increase in the cytosolic Ca^2+^ level is one of the earliest events in stress triggered signaling in plants [5]. Majorly three Ca^2+^ sensors have been involved in sensing these variable levels of Ca^2+^ namely; calcineurin B-like proteins (CBLs), calmodulins (CaMs)/calmodulin-like proteins (CMLs), and calcium-dependent protein kinases (CDPKs) [6], [7]. Among these, CBLs and CDPKs are plant specific Ca^2+^ sensor proteins whereas, CaM is a ubiquitous Ca^2+^ binding protein found in the most eukaryotes [8]. Upon sensing and binding with Ca^2+^, CaM undergoes conformational changes and binds with diverse target proteins, including transcription factors, ion channels/pumps, protein kinases, and phosphatases [8]. Interaction of CaMs/CMLs with their targets modulate several cellular, metabolic, and physiological processes [9], [10], [11]. CAMTAs are the key transcription factors which binds to CaM and regulate Ca^2+^-dependent cellular functions in plants [12], [13]. CAMTAs play a crucial role in plant growth, development, and in stress responses [14], [15], [16]. CAMTAs were first identified in tobacco (NtER1), and they are known by other names also, like signal responsive (SR) and CaM-binding proteins (CaMBP) [18], [19], [17]. CAMTAs bind to a specific 6-bp DNA elements[(A/C)CGTGT or (G/A/C)CGCG(C/G/T)] in their target genes promoter[16]. CAMTAs exist as multigene family in different plants. A total of six CAMTA members have been identified in *Arabidopsis thaliana* [20], seven members in rice(*Oryza sativa*) [9], nine in maize (*Zea mays*) [21], seven in *Medicago truncatula* [22], seven in tomato (*Solanum lycopersicum*) [15], 10 in grapes (*Vitis vinifera*) [14], six in *Camellia sinensis* [6], and four in *Cucumis sativus* [23], [24]. *AtCAMTAs* have been highly responsive to environmental cues, hormonal signals, developmental processes [10], [24]. *AtCAMTA3* has been implicated in the cold stress response where it binds to CBF2 promoter and positively regulates its expression [25]. Overexpression of a soybean *CAMTA* gene, *GmCAMTA12* resulted in enhanced drought stress tolerance in soybean and Arabidopsis [26]. Study of knockout *camta1* mutant revealed that Arabidopsis CAMTA1 positively regulates the drought stress tolerance [27]. Similarly, SlSR1L, a homolog of AtCAMTA1 enhances drought stress tolerance in tomato [28]. Knockout mutation in CAMTA3/AtSR1 decreases ABA sensitivity and enhanced plant’s susceptibility to drought stress. In contrast, overexpression of AtSR1 increases ABA-regulated stomatal closure and imparts drought stress tolerance [29]. The most significant work for understanding the functional role of CAMTA family in plant stress response has been done largely in Arabidopsis. Understanding of the functional mechanisms of CAMTA transcription in abiotic stress signaling and development in important legume crop chickpea is negligible.

In this study, a global investigation was done to identify the *CAMTA* gene family in chickpea. Various features of chickpea CAMTA family were studied, including gene and protein structure, phylogenetic relationship, subcellular localization, and promoter analysis. Protein homology modeling was performed to get an insight into the three-dimensional (3-D) structure of CAMTA protein in chickpea. Expression pattern of CAMTA family was analysed in various developmental stages in chickpea using RNA-seq data. Spatio-temporal expression pattern was studied by RT-qPCR to comprehend the role of chickpea CAMTA family in abiotic stress tolerance. In addition, possible target genes (regulon) of CaCAMTAs were identified.

## 2 Material and methods

### 2.1 Exploration of *CAMTA* genes in chickpea

The Arabidopsis information resource (TAIR) database and Rice Genome Annotation Project-7 (RGAP-7) databases were used to retrieve the protein and nucleotide sequences of *Arabidopsis thaliana* and rice CAMTAs, respectively. These nucleotide and proteins sequences were used as queries to perform homology search at NCBI. The Pfam database (http://pfam.xfam.org/) was used to retrieve Hidden Markov Model (HMM) profiles for CAMTAs. BLAST search was performed against the chickpea proteome using HMM profile. Different databases, including Pfam, Interpro, SMART, and Prosite, were used for the domain structure analysis.

### 2.2 Phylogenetic analysis

The phylogenetic tree was made for investigating the evolutionary relationships between CAMTAs from chickpea and other species. The protein sequences of CAMTAs from various plant species, including moss, lycophytes, angiosperms, and woody tree were retrieved. These sequences were used for multiple sequence alignment (MSA) using ClustalW2. The MSA file was used for generating a neighbor-joining phylogenetic tree with default parameters in MEGAX software. For better visualization, different clades of CAMTAs were marked with various colors using the iTOL web-server [30]. The chromosomal position and homology with Arabidopsis CAMTAs served as the basis for the nomenclature of the *CAMTA* genes in chickpea. The names of the identified genes began with CaCAMTA, followed by a number indicating the corresponding Arabidopsis ortholog. Information about gene features, including locus ID, CDS, intron numbers, and protein size were obtained from NCBI, while protein attributes, like molecular weight (MW) and isoelectric point (pI) were retrieved from ExPASy database.

### 2.3 Gene and protein structure analysis

The open reading frame (ORF) and genomic sequences of CaCAMTAs were obtained from the NCBI database. These sequences were submitted in Gene structure Display Server 2.0 to for creating gene structure model. InterPro was employed to study the protein structure. Protein domain structures were generated using Illustrator for Biological Sequences (IBS) tool.

### 2.4 Protein homology modeling for three-dimensional structure prediction

Advanced homology-based protein modeling programs, such as SWISS-MODEL (http://swissmodel.expasy.org) and Phyre2 [31] were harnessed for predictions of three-dimensional (3-D) protein structures of CaCAMTAs. These programs employ an array of cutting-edge tools, like HHBlits, PSIPRED, and HHSearch to create precise HMM profiles from the query protein sequences. The obtained HMM profiles were used to scan NCBI protein database, and structurally similar proteins with known 3-D structures were identified. Using the identified templates as guide, the protein backbones were modeled. Further, the side chains and loops were incorporated to ensure a comprehensive representation of the protein's spatial arrangement. To further enhance the accuracy of the predictions, advanced ab-initio folding simulations were employed. This allowed the exploration of various conformational possibilities, and refining the model's final structure.

### 2.5 Cloning for subcellular localization

Desi chickpea (ICC4958) cDNA, and gene specific primers were used for PCR amplification of selected *CaCAMTA* genes. The amplicon was inserted into the p-ENTR-D-TOPO vector. To create the YFP fusion constructs of *CaCAMTAs*, their ORFs were mobilized into a destination vector pSITE3CA using the LR recombination protocol. The YFP fusion constructs were verified by PCR and sequencing.

### 2.6 Subcellular localization and confocal microscopy

Subcellular localization of CaCAMTA proteins was performed according to Kamali et al. [32]. Briefly, YFP construct plasmids was used to transiently transform *Agrobacterium tumefaciens* (GV3101:pMP90) cells. Transformed Agrobacterium cells were injected in 5–6-week-old *Nicotiana* leaves. After injection plants were kept in controlled conditions for 48–72 h. Transformed leaves were analysed under confocal microscope (TCS SP5, Leica, Germany) according to Deepika et al. [33].

### 2.7 Promoter analysis

To explore the *cis*-regulatory elements, promoter analysis was performed for CaCAMTAs. From NCBI, 2 kb upstream sequence from translational start point of CaCAMTAs were retrieved. With the use of the Plant CARE database (http://bioinformatics.psb.ugent.be/ webtools/plantcare/html/) [34], the 2 kb upstream sequences were analysed for various *cis*-regulatory elements and motifs.

### 2.8 Expression analysis in different tissues and developmental stages

To investigate the expression patterns of *CaCAMTAs* during various developmental stages, RNA-seq data was retrieved from the NCBI (SRA, ID: SRP121085). The raw reads were processed using the FASTP tool. An index of references genome was built and the raw reads were mapped on the reference genome using HISAT2 [35]. String Tie [36] was used to compile the aligned sequences into potential transcripts, and transcript abundance was calculated as fragments per kilobase of transcript per million reads (FPKM) values (Deepika et al., 2022). The expression profiles were developed for 27 tissues of widely used desi chickpea variety ICC4958. These tissue were the part of various developmental stages, like germination (radicle, plumule and embryo), seedling (epicotyl and primary root), vegetative (root, petiole, stem and leaf), reproductive (Petiole, stem, nodules, root, flowers, buds, pods, immature seeds and leaf), and senescence (immature seeds, mature seeds, seed coat, stem, petiole, root, nodules, leaf and yellow leaf). The log_2 FPKM values were utilized for generating a heat-map employing versatile matrix visualization and analysis software. Furthermore, RNA-seq data from SRA datasets SRP072563 and SRP072564 were extracted to study the specific expression in different seed developmental stages (S1–S7) in distinct seed size desi chickpea varieties (small seeded Himchana 1 and large seeded JGK3).

### 2.9 Plant growth conditions and abiotic stress treatment

ICC4958 desi chickpea variety was utilized for abiotic stress related gene expression study. Seeds were sterilized and seedlings were grown as described by Sagar et al. [37]. Different stress regimes were imposed to ten-day-old chickpea seedlings. For drought stress, seedlings were air-dried. Samples were collected after 0 h, 3 h, 6 h, and 12 h of imposing the drought. For salt stress, roots of seedlings were submerged in a 150 mM NaCl solution. Salt treated samples were harvested after 0 h, 3 h, 6 h, and 12 h. The ABA treatment was given to seedlings by incubating in 100 μM ±ABA solution. ABA treated samples were harvested after 0 h, 3 h, 6 h, and 12 h. Untreated seedlings from the corresponding time periods were taken as controls for various abiotic stress treatments. Stress treated root and shoot tissues were promptly flash-frozen in liquid N_2_.

### 2.10 Total RNA isolation and cDNA preparation

TRIZOL reagent (Ambion) as was used to extract total RNA from 100 mg tissue (root and shoot) per manufacturer's instructions. Extracted RNA was treated with DNase-I to remove DNA impurities. To remove any contaminating chemicals, the RNA was further cleaned by RNeasy Min-Elute Clean-up Kit (QIAGEN). Using a nano-spectrophotometer, the quantity and quality of RNA were verified. 1.2 % denaturing agarose gel was run in MOPS buffer, to ensure the RNA's integrity. The cDNA was prepared using 1 g of pure RNA by high-capacity archive cDNA Synthesis kit.

### 2.11 Expression analysis by RT-qPCR

PRIMER EXPRESS SOFTWARE was utilized for designing RT-qPCR primers for all the genes according to Singh and Pandey [38]. Additionally, primers were examined for self-complementarity. The list of primers is given in Table S1. Three biological replicates of each sample were taken to evaluate the expression in Bio-Rad CFX96 real-time PCR equipment using iTaq Universal SYBR green (Bio-Rad) as described by Sagar et al. [2].

### 2.12 Identification of CaCAMTAs regulon

The prediction of the putative target genes (regulon) regulated by CaCAMTA transcription factors was performed according to Yang and Poovaiah, [39]. To investigate the promoter sequences, entire chickpea genome was extracted from NCBI. The CAMTA transcription factors bind selectively to the CGCG box located in the target gene's promoter region [40], [39]. To identify CGCG box, promoter sequences (2 kb upstream) were retrieved using BED Tools (v2.27.1) with the *getfasta* function. In addition, the Plant Pan (https://plantpan.itps.ncku.edu.tw/) and the Plant Transcription Factor Databases (PTFD) (https://planttfdb.gao-lab.org/) were explored to scan the chickpea genome for the putative target genes containing CGCG box with p-value ≤ 1e-4. The promoter region was further scanned for the CAMTA binding motif by FIMO tool (v5.0.3). The genes with the CGCG box were considered putative targets of CaCAMTAs. Furthermore, to shortlist some potential targets of CaCAMTAs the interactions between CaCAMTAs and their putative target genes were analysed using the PTFD tools (https://planttfdb.gao-lab.org/). The gene IDs of putative target genes were obtained by FIMO tool. Genes IDs of CaCAMTAs and putative targets were used as input in PTFD, and FunTFBS (retrieve regulations among them) method was selected to retrieve potential TF-target interactions with significant p-values.

### 2.13 Statistical analysis

To establish statistical significance, three biological replicates were analysed for all quantitative and expression analyses. Data are shown as the mean over three replicates ± standard deviation (SD). To ensure the statistical significance of the data a two-tailed student's *t*-test was performed. P-values of < 0.05 is considered as statistically significant. The level of significance was indicated by * for P value < 0.05, ** for P value < 0.01, and *** for P value < 0.001.

## 3 Result and discussion

### 3.1 Organization and phylogenetic analysis of CAMTAs in chickpea

**Fig. 1:**
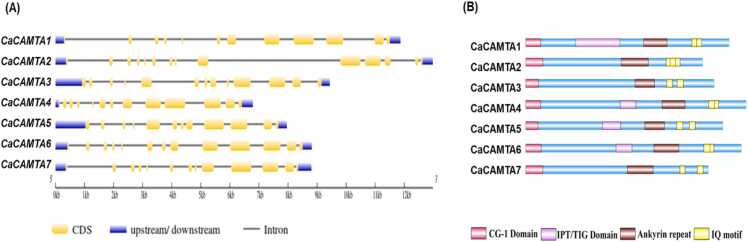
Gene and protein structure of chickpea CAMTA family. (A) Exon–intron organization is shown for *CaCAMTA* genes. Exons, untranslated regions (UTR), and introns are indicated by different color codes. (B) Protein domain structure of CaCAMTA members, they contain conserved DNA binding CG-1 motif, TIG motif, Ankyrin_repeat(Ank_rpt) domains, and IQ motifs.

**Fig. 2:**
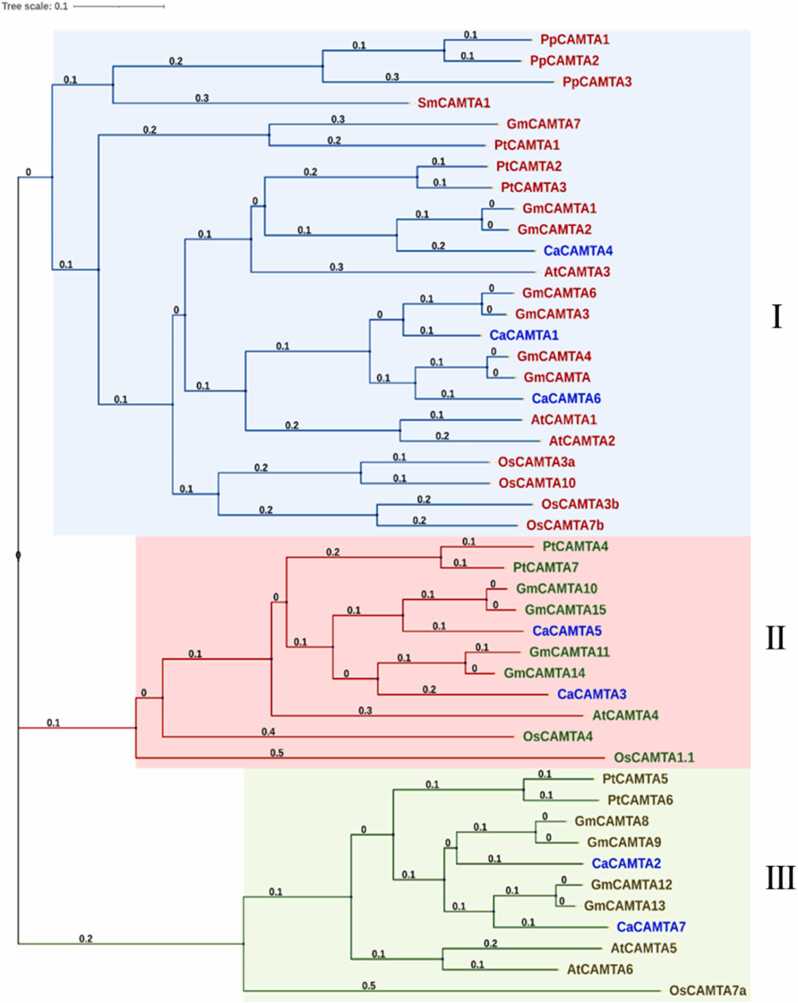
Phylogenetic tree of CAMTAs from chickpea and other plants. Evolutionary relationship between CAMTAs from different plants is shown using phylogenetic tree generated by Neighbor-Joining method using MEGAX software. Chickpea CAMTAs along with their orthologs are divided into three groups: group I, group II and group III. Tree scale bar 0.1 represents amino acid substitution/site*.* At -*Arabidopsis thaliana*, Os-*Oryza sativa*, Gm-*Glycine max*, Pp-*Physcomitrella patens*, Sm-*Selaginella moellendorffii* and Pt-*Populus trichocarpa*.

An exhaustive and thorough homology and sequence-based exploration of databases identified a total of seven *CAMTA* genes in chickpea. These genes were named as *CaCAMTA1* to *CaCAMTA7* based on their homology with Arabidopsis CAMTAs, and chromosomal distribution in chickpea. *CAMTAs* exists as multigene family in different plants. For example, six *CAMTA* gene have been reported in *Arabidopsis thaliana* [20], 15 in wheat (*Triticum aestivum* L.) [41], seven in rice [9], seven in tomato [15], and seven in finger millet (*Eleusine coracana* L.) [42]. Gene structure analysis revealed that *CaCAMTAs* contain multiple exons and introns. The number of introns in *CaCAMTA* genes varied from 11 to 13. *CaCAMTA2* had 13 introns, *CaCAMTA3*, - *4*, -*6* and -*7* had 12 introns each, and *CaCAMTA1* and *CaCAMTA5* had 11 introns each (Fig. 1**A**). A similar pattern of intron-exon arrangement has been observed in different plants. For example, rice *CAMTAs* contain 9–12 introns [9], and wheat CAMTAs had 11–13 introns [41]. This suggests that plant *CAMTAs* are structurally conserved. Domain study revealed that CaCAMTAs harbored the crucial domains, such as the CG-1 box (DNA-binding domain), Ankyrin repeat (ANK_rpt) domain, TIG (Ig-like, transcription factor immunoglobulin), and calmodulin-binding IQ motifs (Fig. 1**B**). Notably, TIG domain was absent in CaCAMTA2, CaCAMTA3, and CaCAMTA7 whereas, all other CaCAMTAs contain a TIG domain. TIG domain has been absent in CAMTAs of several plant species, and these CAMTAs are called as non-TIG-CAMTAs [43]. The non-TIG-CAMTAs were first appeared in the flowering plants, after diverging from non-flowering plants. In other words, when plants adapted to their surroundings, their amino acid sequences changed that possibly resulted in elimination of the TIG domain [10], [43], [24]. Whereas, TIG domain are usually present in CAMTAs of flowering plants. TIG domain facilitates the non-specific DNA binding, and it also affects the protein dimerization [44], [10], [45]. This might affect various target genes and associated biological processes [43]. Interestingly, TIG domain has been found in three CAMTAs in moss, and in a unique CAMTA in lycophyte [43]. It implies that non-TIG-CAMTAs might have evolved recently in angiosperms after diverging from non-flowering plants. The protein size of the seven CaCAMTAs was variable and it ranged from 901aa (CaCAMTA2) to 1102aa (CaCAMTA4). The molecular weight (MW) of CaCAMTAs varied between 102 kDa to 126 kDa (Table 1). Most of the CaCAMTA proteins (excluding CaCAMTA2 and CaCAMTA7) had an isoelectric point (pI) below 7 indicating that chickpea CAMTAs might function optimally in similar microenvironment. A phylogenetic tree was produced to comprehend the evolutionary relationship of chickpea CAMTAs with that of other plants. Protein sequences of CAMTAs from Arabidopsis, Rice, soybean (*Glycine max*), *Populus trichocarpa*, *Selaginella moellendorffii*, *Physcomitrella patens* and Chickpea were used for this analysis (Table S2). Based on significantly high bootstrap values, the phylogenetic tree could be divided into three major clades namely; group I, II, and III (Fig. 2). CaCAMTA1, 4 and 6 belong to group I, CaCAMTA3 and 5 belong to group II, and CaCAMTA2 and 7 belong to group III. CAMTAs from lower species, such as *Selaginella moellendorffii*, *Physcomitrella patens* were grouped together, and appeared earlier in the evolutionary tree, suggesting the early evolution of CAMTAs in lower plants. Interestingly, all the CaCAMTAs were found to be closely associated with soybean CAMTAs. Whereas, they were placed distantly from CAMTAs of monocot plant rice. This suggests that CaCAMTAs have co-evolved with their orthologs from dicot leguminous plants, whereas they have been diverged from monocot plants.

**Table 1:** Details of molecular features of CAMTA genes in chickpea.

**Gene name**	**NCBI Id**	**NCBI Identifier**	**Chromo** **-some**	**Start**	**End**	**Introns**	**CDS Length** **(Bases)**	**Protein** **Length(aa)**	**Isoelectric point (PI)**	**Protein weight (kDa)**
*CaCAMTA 1*	**LOC101514494**	XP_004485582.1	Chr 1	1049607	1061475	11	3071	1023	5.65	115.28
*CaCAMTA 2*	**LOC101493676**	XP_012573334.1	Chr 1	17402638	17415621	13	2705	901	7.02	102.25
*CaCAMTA 3*	**LOC101503411**	XP_012571817.1	Chr 5	42521246	42530680	12	2861	953	5.97	106.98
*CaCAMTA 4*	**LOC101508254**	XP_004504077.1	Chr 6	8086719	8093508	12	3308	1102	5.61	126.02
*CaCAMTA 5*	**LOC101505145**	XP_004504403.1	Chr 6	10864037	10871997	11	2981	993	5.84	110.78
*CaCAMTA 6*	**LOC101498639**	XP_004504800.1	Chr 6	14811032	14819849	12	3245	1081	5.42	121
*CaCAMTA 7*	**LOC101508075**	XP_027192507.1	Chr 7	4551424	4560229	12	2777	925	8.22	105.1

### 3.2 Three-dimensional structure of CaCAMTA proteins

**Fig. 3:**
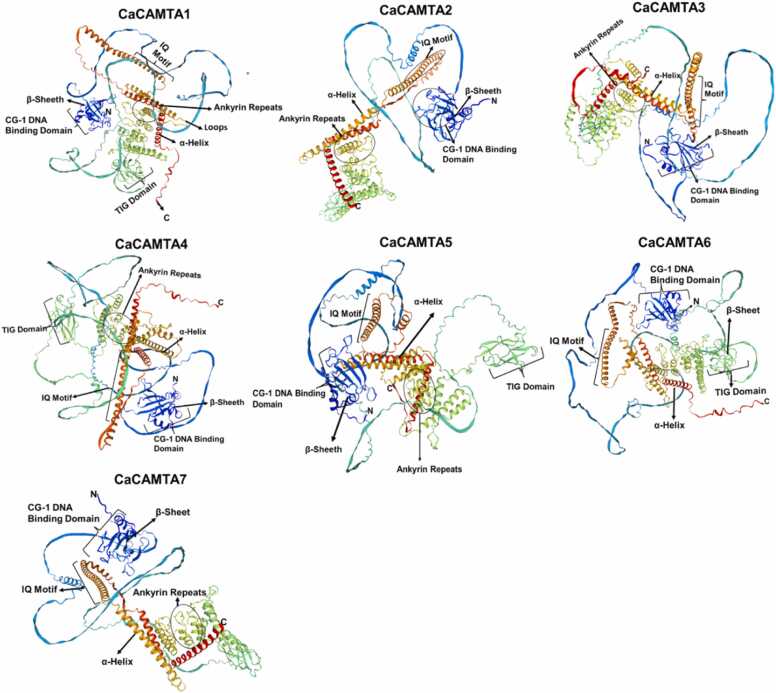
Three-dimensional (3-D) structure of CaCAMTA proteins. 3-D structure was generated for all chickpea CAMTA members using PHYRE2 and SWISS-MODEL. Each CaCAMTA protein is made up of variable number of α-helix, β-strands, loops, IQ motifs, CG-1 DNA binding domain. Except CaCAMTA2, CaCAMTA3 and CaCAMTA7, other CaCAMTA members contain a TIG Domain.

Homology-based protein modeling was done for all the CaCAMTAs to gain insight into their 3-D structure. Protein structures of CaCAMTAs were modeled with 92–99 % confidence, 96–99 % coverage, and 73.83–81.73 % sequence identity. Variable numbers of α-helix, β-strands, disordered regions, loops, and coils were present in CaCAMTAs (Table S3). The β-strand contributed 5–6 %, disordered amino acids contributed 37–48 % whereas, α-helix contributed 22–30 % in a CaCAMTA protein. At the N-terminal, all CaCAMTAs had two antiparallel β-sheets. The CG-1 domain found at the N-terminal of all CaCAMTA proteins, makes a sandwich conformation containing a β-strands in the center. Variable numbers of α-helices are on either side, and loops of varying length are connecting them (Fig. 3). The CG-1 domain binds to the promoters of target genes containing the CG(C/T)G sequence [46]. Particularly, it is involved in regulating the expression of stress related genes [44], [46], [20]. In this study, four CaCAMTAs (CaCAMTA1, 4, 5, and 6) which were composed of several antiparallel β-sheets, and a TIG-domain, classified as TIG-type CAMTAs. Whereas, due to absence of a TIG domain, three CaCAMTAs (CaCAMTA2, CaCAMTA3 and CaCAMTA7) were categorized as non-TIG-CAMTAs. Ankyrin repeat motif was found in all CaCAMTA protein [47]. Ankyrin (ANK) repeats are helix-turn-helix structures, and they have been involved in protein folding, protein stability, and protein-protein interactions. They are also implicated in cell-cell signaling, cytoskeleton integrity, development, and transport mechanisms [48], [49], [50]. All CaCAMTA proteins were composed of a long α-helix, and contained two IQ motifs at the C-terminal (Fig. 3). IQ motifs have been shown to bind to CaM/CML proteins independently of Ca²⁺ [24]. The conformational arrangement of CaCAMTA proteins was like their orthologs in other plants [44], [46], [50], [24]. However, in-depth analysis of the CaCAMTA proteins will provide insights into their functional mechanism.

### 3.3 Subcellular localization of CaCAMTA proteins

**Fig. 4:**
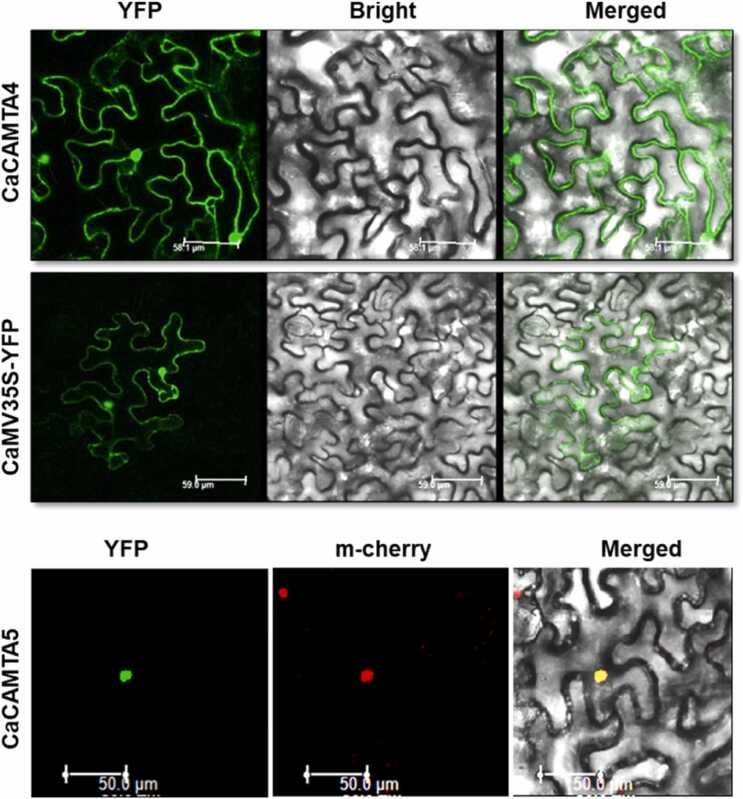
Subcellular localization of CaCAMTA proteins. Expression of YFP-fusion protein in *Nicotiana benthamiana* leaves shows that CaCAMTA4 localized in the nucleus and in the cytosol. CaCAMTA5 was localized in the nucleus. Co-localization was done for CaCAMTA5-YFP with nucleus marker m-cherry to confirm its nucleus localization. Scale bar = 50 μm.

Since we identified the chickpea *CAMTA* genes and their sequences for the first time, it was important to ascertain that they code for the functional proteins. Thus, out of seven *CaCAMTAs* we randomly selected two genes namely, *CaCAMTA4* and *CaCAMTA5*, and their YFP-tagged proteins were transiently expressed in *Nicotiana benthamiana* to analyse their subcellular localization. Using confocal microscopy, the yellow fluorescence of YFP fused CaCAMTA4 protein was detected in the nucleus and the cytoplasm. CaCAMTA5 was localized exclusively in the nucleus, which was confirmed by co-localization of YFP fluorescence of CaCAMTA5 with the red fluorescence of nucleus marker m-cherry (Fig. 4). This analysis confirmed that the identified CaCAMTA genes code for the functional CAMTA proteins. Moreover, localization of CaCAMTA proteins in the nucleus is appropriate for their role as transcription factors. Like other TFs, CAMTAs harbor nuclear localization signal (NLS) sequence which facilitate the entry of CAMTAs in the nucleus. However, in Arabidopsis CAMTAs have been shown to localized in cellular compartments other than nucleus[51], [52]. It is possible that some CAMTA protein may normally reside in the extranuclear space, but they may translocate to nucleus in response to specific stimuli like environmental stresses.

### 3.4 *Cis*-regulatory elements in *CaCAMTA* promoters

**Fig. 5:**
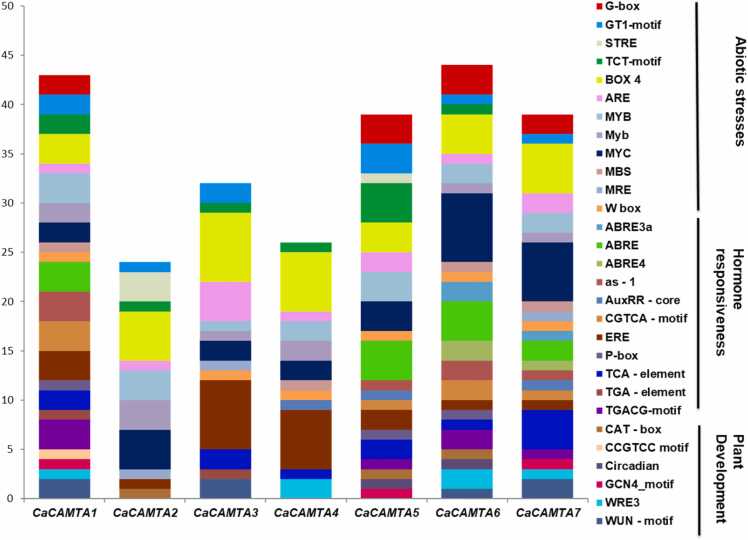
*Cis*-regulatory elements in the promoters of *CaCAMTAs* genes. Bars of different colors indicate *cis*-regulatory elements 2 kb promoter of *CaCAMTA* genes. On the X-axis, name of the gene is indicated whereas, on the Y-axis number of *cis*- regulatory elements in *CaCAMTA* promoters is marked.

The transcription level of a gene is largely determined by the *cis*-regulatory elements in its promoter. Thus, to understand the regulation of *CaCAMTA* genes, their promoters were analyzed for the *cis*-regulatory elements. Various elements were discovered in 2 kb upstream promoter of *CaCAMTA* genes. Identified *cis*-regulatory elements could be classified into three main categories i.e., development related, phytohormone response related, and abiotic stress related (Fig. 5). *CaCAMTA1* and *CaCAMTA6* promoters were found to have the highest, 43 and 44 *cis*-regulatory elements, respectively. *CaCAMTA2* was found to have the least, only 24 *cis*-regulatory elements. These included salt stress, ABA, and drought responsive *cis*- regulatory elements, like TCA element, ABRE, ABRE3a, ABRE-4, MBS, MYB, MYC, and TC-rich repeats [53]. The promoters of all *CaCAMTAs* featured a substantial number of light responsive' motifs, such as G-box, TCT motif, Box4, and others (Table S4). The presence of abiotic stress related *cis*- regulatory elements implies that different TFs may bind and regulate the expression of *CaCAMTA* genes under various abiotic stresses. *CaCAMTA1, 5, 6*, and *7* promoters contained the ABRE motif along-with abiotic stress responsive motifs, like MYB and MYC, indicating that these CaCAMTAs might regulate abiotic stresses through ABA-dependent pathway. It is well-known that ABA serves as a connecting point of diverse developmental processes and abiotic stress responses [54]. Particularly, during seed development and maturation, dehydration is prompted that causes seed dormancy [55]. Furthermore, most of the *CaCAMTA* promoters contained hormones response-related *cis*- regulatory elements, such as Aux RR-core (auxin response), ERE (ethylene response), and CGTCA & TGACG (Jasmonic acid response) [32]. Notably, *CaCAMAT3* and *CaCAMAT4* contained the highest number (6 and 7, respectively) of ERE motifs. The presence of the ERE and other hormone related *cis*-regulatory elements in majority of the *CaCAMTA* promoters underpins the involvement of *CaCAMTAs* in plant development, and environmental stresses responses. Thus, *CaCAMTAs* may mediate the interaction of stress signaling and hormone signaling via various *cis*-regulatory elements.

### 3.5 Expression profiles of *CaCAMTA* genes during development

**Fig. 6:**
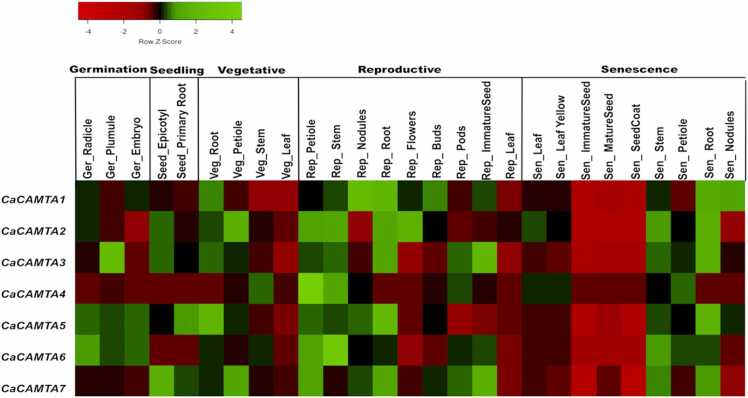
Expression pattern of *CaCAMTAs* during chickpea developmental stages. The expression pattern of *CaCAMTA* genes during various developmental stages is shown by the heat-map. These stages include germination stage (24–36 h post imbibition), seedling stage (8–10 days post germination), vegetative stage (20–25 days post germination), reproductive stage (40–50 days post germination), and senescence (90–110 days post germination). Name of genes are mentioned on left side. Different tissues, and developmental stages are marked at top of the heat-map. The scale indicates normalized log_2 FPKM values.

Expression analysis was done to comprehend the role of *CaCAMTA* genes in chickpea plant development. The analysis covered 27 different tissues belonging to various developmental stages, like germination (24–36 h post imbibition), seedling (8–10 days post germination), vegetative (20–25 days post germination), reproductive (40–50 days post germination), and senescence (90–110 days post germination) (Table S5). The analysis revealed that most *CaCAMTA* genes displayed differential expression pattern in different tissues, across developmental stages. Almost all the *CaCAMTA* genes showed significant induction during senescence stages specifically, in immature seed, mature seed and seedcoat. *CaCAMTA1* was significantly expressed in vegetative stages, including stem and leaf. Strikingly, *CaCAMTA2* expressed in specific tissues, such as embryo during germination stage, vegetative leaf, nodules and pods in reproductive stage, and nodules in senescence stages (Fig. 6). *CaCAMTA3* showed specific expression in embryo during germination, in vegetative and reproductive leaf, and flowers during reproductive stage. *CaCAMTA4* was significantly induced throughout the germination and seedling stages, and in some tissues of reproductive development and senescence. *CaCAMTA5* had a unique expression profile, and it was down-regulated in most tissues of early reproductive development (petiole, stem, nodules, and root). Whereas, it was significantly up-regulated in tissues during later reproductive stage. The involvement of CAMTAs during various developmental stages has been reported in different plants. In tomato, all seven *CAMTA* genes were differentially expressed during various fruit ripening stages [15]. Heterologous expression of *Prunus persica* CAMTA1 (*PpCAMTA1*) in Arabidopsis complemented the defective development phenotype of *camta2* and *camta3* mutant, and reinstated the plant size to WT level [56]. This suggests the involvement of CAMTA in the vegetative development. In peach, CAMTA2 was found to be a key regulator of fruit setting and softening [57]. In Arabidopsis, CAMTA1 and CAMTA5 bind to the promoter of AVP1 (Arabidopsis V-PPase1 gene) and enhances its expression to promote pollen development[58]. Overall, these observations suggest that *CAMTA* genes play crucial roles during different developmental stages in plants. Thus, it can be hypothesized the CaCAMTAs may also regulate plant development in chickpea.

### 3.6 Expression pattern of *CaCAMTAs* during seed development

**Fig. 7:**
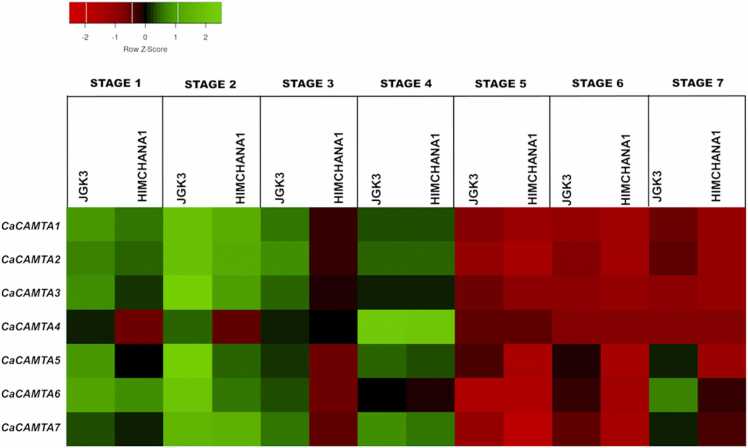
Expression pattern of *CaCAMTAs* during seed development. The expression pattern of *CaCAMTAs* during seed development (S1–S7 stage) in JGK3 and Himchana1 chickpea varieties is indicated by heat-map. Name of *CaCAMTA* genes, chickpea variety and seed stages are marked on the heat-map. The scale bar indicates the log_2 Fold change FPKM values.

The seed development determines the chickpea yield thus, optimum seed development is crucial from economic perspective. To understand the role of CaCAMTAs in seed development, their expression profiles were generated in small-seeded chickpea variety Himchana 1 and large-seeded variety JGK3. The expression profiling was done during crucial stages of seed development in chickpea, including early to late embryogenesis (S1-S4), and mid-maturation to late-maturation (S5–S7) [59] (Table S6). Interestingly, all the *CaCAMTAs* displayed differential expression pattern in both the chickpea varieties. Notably, all the CaCAMTAs were found to be significantly up-regulated during mid-late maturation (S5–S7) stages. Whereas, they were down-regulated in most of the early-mid seed developmental stages (S1-S4) (Fig. 7). *CaCAMTA4* displayed a unique expression pattern though, as it was specifically up-regulated during S1 and S2 stages of Himchana 1, but had insignificant expression in JGK3 during these stages. Similarly, *CaCAMTA5, 6* and *7* were specifically up-regulated during S3 in Himchana 1. Also, the expression level of *CaCAMTA5, 6* and *7* during S5-S7 stages was significantly higher in Himchana 1 than JGK3. This indicates that same set of *CaCAMTA* genes may positively regulate the mid to late maturation stages of seed development, and negatively regulate early to late embryogenesis in both the varieties. Notably, most *CaCAMTAs* show preferentially higher expression in small seeded chickpea Himchana1. Previously, different components of Ca^2+^ mediated signaling, including CIPKs and CDPKs have been found to be induced at similar developmental stages of these chickpea varieties [60], [59]. Like chickpea, rice orthologs of CBLs, CIPKs and CDPK and were significantly induced in different seed stages [61], [62]. This suggests that Ca^2+^ signaling is important for chickpea seed development. Like other Ca^2+^ signaling components, CAMTA transcription factors could be the key regulators of Ca^2+^ signaling triggered gene expression during chickpea seed development.

### 3.7 Expression pattern analysis of *CaCAMTAs* under abiotic stresses and ABA

**Fig. 8:**
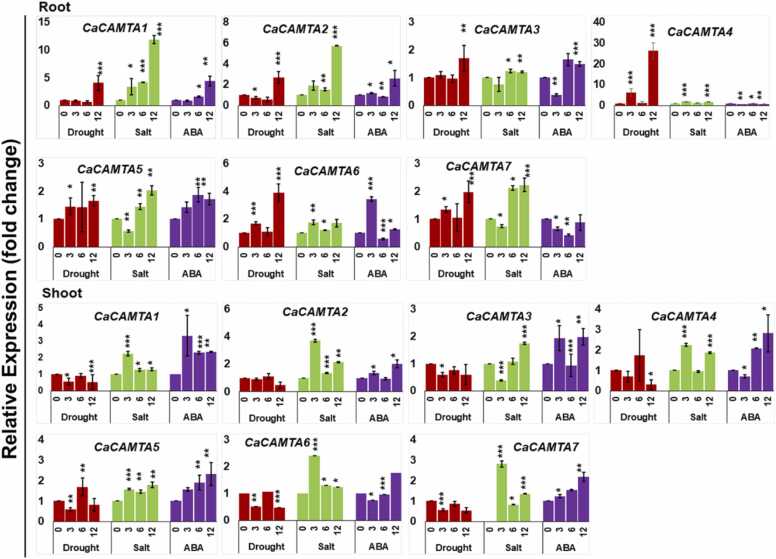
Expression profiles of *CaCAMTAs* under abiotic stress and ABA treatments. The expression profile of *CaCAMTAs* were generated under abiotic stresses like drought, salt, and ABA treatment in chickpea using RT-qPCR. Stress regimes are shown on the X-axis of the graph whereas the relative expression is shown on the Y-axis. Graph bar depicts the average of three replicates. Error bars are indicative of the standard error among the samples. Asterisk * represents p-value < 0.05, ** p-value < 0.01 and *** p-value < 0.001 for treated samples w.r.t. untreated control.

To investigate the expression pattern of *CaCAMTAs* under abiotic stresses, we generated their RT-qPCR based expression profiles under drought, salt stress, and ABA. Expression was studied for all the *CaCAMTA* genes, after 0 h (control), 3 h, 6 h and 12 h of stress treatment. Based on the criteria of the expression fold change value ≥ 2.0 relative to the untreated control, all the *CaCAMTAs* displayed differential expression under drought, salt, and ABA treatments (Table S7). In roots, five *CaCAMTA* genes showed differential expression under drought stress, including *CaCAMTA1, 2, 4, 6* and *7*. Interestingly, all five genes were found to be up-regulated. *CaCAMTA4* was induced at 3 h and 12 h of drought stress whereas, other four *CaCAMTA* genes were up-regulated specifically at 12 h of drought. Four genes, including *CaCAMTA1, 2, 5* and *7* were up-regulated under salt stress. Out of these, *CaCAMTA1* expressed at all time points of salt stress whereas, *CaCAMTA7* expressed at 6 h and 12 h time points. Importantly, three genes *CaCAMTA1, 2* and *7* were induced under both, drought, and salt stress (Fig. 8). Under ABA treatment, only three genes namely *CaCAMTA1*, *2* and *6* were found to be up-regulated, and none of the genes was significantly downregulated in roots. In shoots, contrary to roots, four genes namely *CaCAMTA2, 4, 5* and *6* were down-regulated after 12 h drought stress. Thus, *CaCAMTAs* are activated under drought stress in roots possibly to control the transcription of genes, like water transporters and channels to facilitate the water and nutrient uptake through roots. Under salt stress, except *CaCAMTA5*, all the genes were differentially expressed in shoots. Out of these, *CaCAMTA3* was down-regulated whereas, other five genes were up-regulated. In contrast to roots where *CaCAMTA* genes were induced mostly during later time point (6 h and 12 h) of salt stress, most *CaCAMTA* genes were induced at early stage(3 h) of salt stress in shoots (Fig. 8). The osmotic and Na^+^ ion toxicity effects of salt stress are proposed to be temporally and spatially isolated. Salt induced osmotic/drought stress affects appear early, and that Na^+^-specific responses are induced later [63]. In our study, the expression pattern suggests that *CaCAMTA* genes could variably be involved in regulating the biphasic effect of salt stress. Early inducing *CaCAMTA* genes might be involved in regulating salt stress triggered osmotic/drought stress whereas, late inducing *CaCAMTA* genes might regulate Na^+^ -ion stress related response in chickpea. Under ABA treatment, all *CaCAMTA* genes except *CaCAMTA6* were up-regulated. *CaCAMTA1* was induced at all time points, whereas other genes were mostly induced after 12 h of ABA treatment. Overall, this data suggests that *CaCAMTA* genes could be involved in regulating abiotic stress tolerance via controlling the expression of diverse set of genes in roots and shoots. In roots, they might regulate drought and salt stress related genes which could regulate the uptake of water and nutrients like NO_3_^-^ and K^+^ which are crucial for drought and salt tolerance. Whereas, in shoots, *CaCAMTA* genes might control the expression of Na^+^ compartmentalization and sequestration related genes that may contribute to leaf tissue salt tolerance. In addition, by regulating the ABA signaling, *CaCAMTA* genes might promote ABA mediated stomata closure. Thus, they may contribute drought/osmotic stress tolerance via limiting the water loss through transpiration. Previously, CAMTAs have been shown to regulate abiotic stress tolerance in different plant species. For example, in tomato, *SlSR1/CAMTA* and *SlSR3* express significantly under drought stress. Virus induced gene silencing of *SlSR1* and *SlSR3* led to rapid water loss through leaves, and altered expression of drought stress related genes, consequently reduced drought stress tolerance in tomato [28]. In Arabidopsis, CAMTA6 plays a crucial role in germination stage salt stress tolerance. *CAMTA6* was induced by salt treatment during germination, and the *camta6* mutant displayed increased salt and ABA tolerance, and reduced Na^+^ accumulation at the seed germination stage [64]. In a recent study, CAMTA associated salinity tolerance was shown to be mediated by CML proteins. CML13 and CML14 by interacting with CAMTA proteins regulated salinity stress response in Arabidopsis [65]. It is quite possible that CaCAMTAs may also interact with CMLs and other Ca^2+^ related proteins, and regulate the expression of key drought and salt stress related genes like SOS, HKTs, aquaporins, Na^+^/ K^+^ transporters and channels in chickpea. This opens the avenues for future investigations related to CaCAMTA functions in chickpea. Overall, the expression dynamics of *CaCAMTA* genes hints towards their key role in abiotic stress responses in chickpea. In future, potential candidates will be exploited for genetic manipulation of chickpea plants for enhanced abiotic stress resilience and crop improvement.

### 3.8 Identification of CaCAMTA regulon in Chickpea

Since CAMTAs are TFs, they demonstrate their functional role mainly by transcriptional regulation of their target genes in plants cell. A set of genes which are transcriptionally regulated by a TF constitutes its regulon. CAMTAs have been known to bind at the CGCG box in the promoters of their target genes [39]. Here, we found a total of 1660 genes whose promoters were enriched with CGCG motif (Table S8). These genes are the possible targets of *CaCAMTAs* thus, they represent the CaCAMTAs regulon in chickpea. The CaCAMTA regulon includes orthologous genes to those which are involved in regulating abiotic stress responses for example, heat shock protein and heat shock factor -binding protein (HSBP) [66], [67], late embryogenesis abundant protein D-34, serine/threonine-protein kinase and phosphatases [54], [68]. The regulon also included hormone signaling and plant development related genes, such as abscisic acid receptor PYL4-like, auxin-responsive protein (SAUR36, SAUR32), ABC transporter I family member, gibberellin 3-beta-dioxygenase (GA3OX1) and Aux/IAA genes. In addition, genes related to ion transport (phosphate transporter PHO1 homolog 9), water transport (aquaporin NIP6–1-like), and ROS homeostasis related genes (respiratory burst oxidase homolog protein A, RBOH-A and cytochrome P450) were included in the CaCAMTA regulon (Table S8). Furthermore, with *in-depth* analysis of probable interaction of TFs with their possible targets, we found out total 42 potential targets of CaCAMTAs (Table 2). This set of potential CaCAMTA targets included some key genes, like abiotic stress related dehydration-responsive element-binding protein 1E-like (DREB), protein EARLY-RESPONSIVE TO DEHYDRATION, plasma membrane associated Calcium-transporting ATPase, ABC transporter E family member, and myb like proteins. These targets also included plant development related genes, including ethylene-responsive transcription factor (ERF), abscisic acid-insensitive 5 (ABI5)-like protein, and pollen receptor-like kinase 4. This suggests that members of CaCAMTA family TFs may bind at the promoters of this diverse group of genes, and control their transcription in response to abiotic stress and plant development. This is also consistent with the differential regulation of CaCAMTAs expression under abiotic stresses, ABA treatment and during various developmental stages. Previously, it was reported that CAMTAs may regulate plant growth and development in response to environmental stresses by integrating phytohormones (e.g., auxin, GA, and ABA) signaling[69]. Thus, identification of CaCAMTAs regulon unfolds another layer of CAMTA mediated regulatory mechanism in chickpea. In future, investigation of binding and transcriptional regulation of some of these target genes by CaCAMTAs will provide the crucial insights into their regulatory network and functional role in chickpea.

**Table 2:** List of potential targets genes of CaCAMTAs in chickpea.

**S.No.**	**Target Locus ID**	**Target gene annotation**	**p-Value**
1	LOC101489173	topless-related protein 3-like	9.2E−05
2	LOC101494182	Ethylene-responsive transcription factor ERF017-like	4.4E−05
3	LOC101499269	Cytokinin riboside 5′-monophosphate phosphoribohydrolase	2.9E−05
4	LOC101499463	Calcium-transporting ATPase plasma membrane-type	4.4E−05
5	LOC101500237	Interferon-related developmental regulator 1	4.4E−05
6	LOC101501038	NDR1/HIN1-like protein 10	4.4E−05
7	LOC101501102	Protein antagonist of like heterochromatin protein 1	4.4E−05
8	LOC101507508	Uncharacterized protein LOC101507508	9.2E−05
9	LOC101509376	Oxysterol-binding protein-related protein 3A-like	9.2E−05
10	LOC101509717	Kinetochore protein SPC25 homolog isoform X1	4.4E−05
11	LOC101510445	Protein ALP1-like	4.4E−05
12	LOC101511599	LOB domain-containing protein 41	9.2E−05
13	LOC101514793	Polyadenylate-binding protein RBP47C-like	4.4E−05
14	LOC101514990	Indole−3-acetic acid-induced protein ARG2	9.2E−05
15	LOC101514996	Auxin-induced protein 22D-like isoform X2	1.3E−05
16	LOC101490016	ABC transporter E family member 2	9.2E−05
17	LOC101492009	Protein TIFY 5 A	9.2E−05
18	LOC101493799	Probable Serine/Threonine-Protein Kinase PBL19	9.2E−05
19	LOC101493851	Monoglyceride Lipase	9.1E−07
20	LOC101495575	Protein EARLY-RESPONSIVE TO DEHYDRATION	1.3E−05
21	LOC101495711	Proline-Rich Extensin-Like Protein EPR1	9.2E−05
22	LOC101495842	Probable Serine/Threonine-Protein Kinase PIX13	1.3E−05
23	LOC101496665	Xyloglucan Endotransglucosylase/Hydrolase 2	9.2E−05
24	LOC101496714	Peptidyl-Prolyl Cis-Trans Isomerase FKBP20–1	9.2E−05
25	LOC101499882	Uncharacterized Protein LOC101499882	9.2E−05
26	LOC101500302	Probable WRKY Transcription Factor 70	9.2E−05
27	LOC101500325	Aspartic Proteinase-Like Protein 2	9.2E−05
28	LOC101505186	Dehydration-Responsive Element-Binding Protein 1E-Like	4.4E−05
29	LOC101505929	Pollen Receptor-Like Kinase 4	4.4E−05
30	LOC101507508	Uncharacterized Protein	9.2E−05
31	LOC101509476	myb-like protein AA	9.2E−05
32	LOC101509735	Ubiquitin-conjugating enzyme E2	9.2E−05
33	LOC101512782	Phosphatidylinositol 4-kinase gamma 5-like	2.9E−05
34	LOC101513365	GATA transcription factor 8	9.2E−05
35	LOC101513633	BTB/POZ domain-containing protein	4.4E−05
36	LOC101515285	myb-related protein 306-like	4.4E−05
37	LOC101515709	Protein IQ-DOMAIN 31 isoform X2	9.2E−05
38	LOC101489011	E3 ubiquitin-protein ligase RDUF1-like	1.3E−05
39	LOC101502949	Digalactosyldiacylglycerol synthase	4.4E−05
40	LOC101505449	ABSCISIC ACID-INSENSITIVE 5-like protein 2	4.4E−05
41	LOC101515285	myb-related protein 306-like	4.4E−05
42	LOC101515409	Ubiquitin-conjugating enzyme E2 22	1.3E−05

## 4 Conclusions

In conclusion, the whole complement of *CAMTA* genes have been identified in chickpea. This gene family is highly conserved in terms of gene and protein structures. Promoter analysis and gene expression analysis indicate that *CaCAMTA* genes might regulate abiotic stress tolerance and plant development. Most of the *CaCAMTAs* displayed differential expression under abiotic stresses and during developmental stages. Some of the important genes like *CaCAMTA1*, *4* and *6* which were significantly induced under abiotic stresses, and during seed developmental stages could be the crucial regulators of crop yield under stress conditions. Thus, they could be of biotechnological importance and might be utilized in developing improved chickpea crop. Moreover, identification of CaCAMTA regulon opens the new avenues for functional characterization of important target genes in chickpea. In the future, functional characterization of the key genes will help in deciphering the molecular mechanism of CAMTA mediated stress tolerance in chickpea.

## Funding

Authors are thankful for the financial support from BRIC-NIPGR core research grant.

## CRediT authorship contribution statement

**Amarjeet Singh:** Writing – review & editing, Visualization, Supervision, Resources, Project administration, Investigation, Funding acquisition, Formal analysis, Conceptualization. **Kamankshi Sonkar:** Writing – original draft, Visualization, Software, Resources, Methodology, Investigation, Formal analysis, Data curation. **Saravanappriyan Kamali:** Visualization, Software, Methodology, Formal analysis, Data curation. **Atul Kumar:** Visualization, Software, Formal analysis, Data curation. **Deepika Deepika:** Visualization, Validation, Methodology, Formal analysis. **Ankit Ankit:** Visualization, Validation, Methodology, Investigation.

## Declaration of Competing Interest

The authors declare that they have no known competing financial interests or personal relationships that could have appeared to influence the work reported in this paper.
